# Prevalence of sarcopenia in multi ethnics adults and the association with cognitive impairment: findings from West-China health and aging trend study

**DOI:** 10.1186/s12877-020-1468-5

**Published:** 2020-02-17

**Authors:** Xiaolei Liu, Lisha Hou, Xin Xia, Yang Liu, Zhiliang Zuo, Yan Zhang, Wanyu Zhao, Qiukui Hao, Jirong Yue, Birong Dong

**Affiliations:** 1 National Clinical Research Center for Geriatrics, West China Hospital, Sichuan University, No. 37, Guo Xue Xiang Renmin Nan Lu, Chengdu, Sichuan China; 2 Geriatric Health Care and Medical Research Center, Sichuan University, Chengdu, Sichuan Province China

**Keywords:** Sarcopenia, Aging, Western China, Multi-ethnic, Cognitive impairment

## Abstract

**Background:**

Sarcopenia is a condition that is characterized by loss of muscle mass, muscle strength and muscle functional impairment with ageing. It is associated with poor health outcomes, premature death and a significant burden on the global health economy. The prevalence of sarcopenia in China is unknown since most of the studies are lack of uniform standard. The study was undertaken to study the prevalence of sarcopenia and the association with cognitive impairment among multi-ethnic adults aged 50 years old or older in western China.

**Methods:**

We measured gait speed, handgrip strength and muscle mass by using bioelectrical impedance analysis (BIA) for all eligible participants and 4500 participants were eligible for the analysis. We defined sarcopenia using the diagnostic algorithm recommended by the Asian Working Group for Sarcopenia (AWGS). We assessed the participants’ cognitive functions using the 10-item Short Portable Mental Status Questionnaire (SPMSQ). Relationships between sarcopenia and cognitive impairment were analyzed using univariate and multivariate analyses.

**Results:**

Of 4500 participants (mean age 62.4 ± 8.3 years), 869 (19.31%) adults were sarcopenia. 446 (9.9%) participants were identified as having mild cognitive impairment, 144 (3.2%) adults were identified as having moderate/severe cognitive impairment. After adjusting for age, gender, ethnics and other potential cofounders, cognitive impairment was found to be independently associated with sarcopenia with a dosage effect (mild cognitive impairment: odds ratio [OR]: 1.41, 95% CI 1.10–1.82; moderate/severe cognitive impairment: OR: 3.05, 95% CI 2.08–4.49). After gender stratification, the association between mild cognitive impairment with sarcopenia in male is not significant, while is still significant in female. While the association between moderate/severe cognitive impairment is independently associated with sarcopenia in both male and female.

**Conclusions:**

The prevalence rates of sarcopenia, mild cognitive impairment, moderate/severe cognitive impairment among the communities aged 50 or older in western China were 19.31, 9.9 and 3.2%, respectively. Cognitive impairment was significantly associated with sarcopenia with a dosage effect, especially in female.

## Background

Sarcopenia was first named in 1989, an age-associated loss of skeletal muscle mass and function [1]. The 2010 European Working Group on Sarcopenia in Older People (EWGSOP) defines it as a type of progressive, extensive reduction in skeletal muscle mass, muscle strength and skeletal muscle dysfunction, leading to a decline in body function and quality of life, and even death [2]. It is generally characterized by decreased muscle mass and bone mass with aging, as well as skeletal muscle dysfunction, which in turn affects the normal physiological function and quality of life [3]. In 2016, sarcopenia was officially included in the ICD-10 disease code [2], marking the diagnosis of sarcopenia in the field of clinical medicine, which is considered as a disease with its unique characteristics. Besides, sarcopenia was considered as a mortality predictor in community-dwelling older adults, meaning that older adults with severe sarcopenia had an increased risk of death in the short term [4].

The incidence of sarcopenia reported in different countries varies greatly depending on the method of measurement, the population surveyed, and the diagnostic criteria. Prevalence rates using EWGSOP definition vary from 1 to 29% in elderly community-dwelling populations and from 14 to 33% in long-term care populations in western countries [5]. While the prevalence of sarcopenia in Asia is 2.5–45.7% using Asia Working Group for Sarcopenia (AWGS) definition [6]. However, many challenges remained to be solved in the future. Asia is made up of a great number of ethnicities. The majority of currently available studies have been published from eastern Asia. Therefore, more studies of sarcopenia in the south, southeastern, and western Asia should be promoted.

China is the most populous country in Asia with the world’s largest aging population. By 2050, there will be 400 million Chinese citizens aged over 65 years old, and 150 million of whom will be over 80 years old [7]. Besides, China is a multi-ethnic country with 56 ethnicities. The prevalence of sarcopenia in multi-ethnic western China is unknown. Screening and assessment of sarcopenia may provide an opportunity for early detection, intervention, and monitoring of the most vulnerable Chinese elders to reduce morbidity, prevent disability, and enable more effective use of health care resources in minority areas. Besides, although the association between sarcopenia and adverse health outcomes has been described, the interrelationship between cognitive impairment and sarcopenia in older people remains a controversy.

In this study, we got the cross-sectional data from the West-China Health and Aging Trend (WCHAT), a longitudinal multi-center cohort research conducted in western China to assess the health and aging status in the multi-ethnic region. According to the diagnostic algorithm of AWGS, participants with low muscle mass as well as low muscle strength or physical performance were considered to have sarcopenia. We aimed to 1) evaluate the prevalence of sarcopenia according to the recommended criterion of the AWGS. 2) investigate the association between cognitive impairment and sarcopenia.

## Method

### Participants

**Fig. 1:**
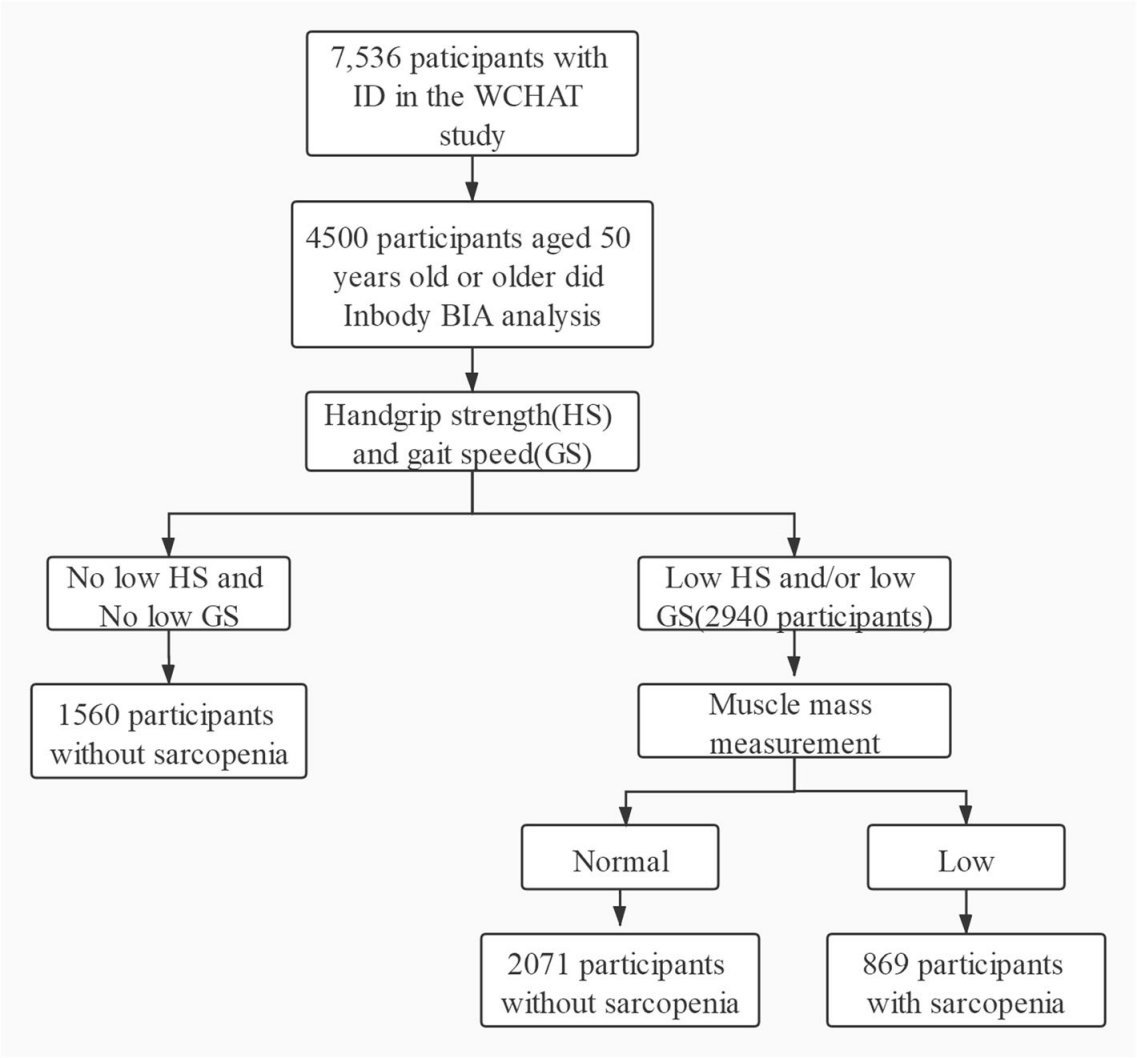
Flow chart of study participants. Initially a total of 7536 community-dwelling multi-ethnics Chinese aged 50 or older were recruited, 4500 participants did bioelectrical impedance analysis (BIA) were analyzed in this study. Among them, 1560 participants without low gait speed and low grip strength, 2071 participants without low ASMI and 869 participants met the AWGS criteria of sarcopenia

The current research is a cross-sectional analysis obtaining baseline data of the WCHAT study collected from July to December in 2018. Data were collected from 4 provinces including Yunnan, Guizhou, Sichuan, and Xinjiang. All participants aged 50 years old or older were enrolled. Participants were recruited by convenience and asked verbally by the researchers about their willingness to take part in the study. Before investigation, informed consent was signed and obtained by each participant. Initially, we recruited 7536 community-dwelling multi-ethnic Chinese aged 50 and older in total. 4500 participants did the bioelectrical impedance analysis (BIA) which is available for the selection of sarcopenia (Fig. 1).

### Data collection

All interviewers are medical students in Sichuan University and was trained for 2 days about how to collect questionnaire data through face to face, one-on-one personal interviews. Other anthropometric and bioimpedance measurements were collected by trained technicians.

### Sarcopenia screening

Sarcopenia was measured by the recommended diagnostic algorithm of AWGS in which included three elements: muscle mass, muscle strength, and physical performance. Muscle mass was measured by the method of BIA and this has been verified reliability and validity in Chinese [8]. The BIA method adopted the INbody720 body composition instrument, which was widely used in the diagnosis of sarcopenia [5, 9, 10]. Previous research did not observe statistical difference between MRI-measured and BIA-derived SM [11]. And the cut-off value of appendicular skeletal muscle mass index (ASMI) was 7.0 kg/m^2^ in men and, 5.7 kg/m^2^ in women. While grip strength was used a dynamometer (EH101; Camry, Zhongshan, China) to test the muscle strength. During the test, subjects held the grip dynamometer with their dominant hand, stood upright, kept their feet naturally separated (shoulder-width apart) and their arms could naturally droop. At the beginning of the test, the subjects gripped the handle to their full capacity. Intermittent gripping, swinging of the arms, squats, or contact with other body parts was prohibited. Test were performed on two independent occasions and the largest value was recorded. And the cut-off value was 26 kg in men and,18 kg in women [12]. Usual walking speed was measured. Subjects stood at the starting point and upon the starting command, walked forward at a normal pace to the 4-m line. The walking time was recorded using a kind of infrared sensor and the acceleration phase was strictly excluded. During the test, subjects wore common shoes, could use mobility aids, but could not be assisted. There were no time limits to the assessments and subjects could stop and rest if necessary. Sitting down was prohibited. And for sarcopenia definition, the cut-off value of gait speed was 0.8 m/s [5].

### Demographic information

Demographic information included age, gender, ethnics, marriage status, living status and education levels.

### Daily lifestyles and chronic diseases

Smoking, alcohol drinking, and tea drinking habits were used to assess the daily lifestyles of the participants. Smoking status was categorized as smoking and no smoking. Alcohol and tea drinking status was categorized as drinking and no drinking. Sleep quality was assessed using Pittsburgh sleep quality index (PSQI). Scores > 5 are considered as poor self-reported sleep quality which was sensitive in distinguishing good vs. poor sleepers in Chinese [13]. Indoor housework and outdoor housework were also asked by the interviewers. A medical history of chronic disease was self-reported by the participants or their caregivers. These disease conditions included hypertension, coronary heart disease, chronic obstructive pulmonary disease, osteoarticular disease, tumor, stroke, diabetes mellitus, cataract, deaf and so on. Chronic diseases comorbidities were considered as having two or more kinds of chronic diseases.

### Cognitive function, depressive symptoms and anxiety status

Cognitive status was measured using a 10-item Short Portable Mental Status Questionnaire (SPMSQ). For SPMSQ scoring, 0~2 indicated complete cognitive function, 3~4 indicated mild cognitive functional impairment, 5~7 indicated moderate cognitive function impairment, and 8~10 indicated severe cognitive function impairment. And this judgment was based on the educational level [14]. Depressive symptoms were assessed using the 15-item Geriatric Depression Scale (GDS-15). The scale, which contains 15 items that require only a yes/no answer, is the most widely used scale for the detection of depression. GDS-15 scores ≥5 indicate depressive mood [15]. Anxiety status was assessed using Generalized Anxiety Disorder (GAD-7) questionnaire and the scores ≥5 was considered as having anxiety status [16].

### Statistical analysis

We test the normality of variables using R version 3.6.1. The measurement data is expressed by X ± SD. For the normal distribution variables, the difference between the groups is compared by the independent sample T-test and the count data is expressed in %, using the χ2 test. We used χ2 test to determine whether the prevalence of cognitive impairment related factors was differed by sarcopenia status. Associations with a *p*-value of 0.1 or less in the univariate analysis were selected for the multiple regression analysis, which were used to estimate the odds ratio (OR) to identify associations between sarcopenia and cognition impairment after adjusting for potential confounders. A value of P<0.05 (two-side) was considered to be statistically significant.

## Results

Overall, we enrolled 4500 participants (1627 men and 2873 women) aged 50 years old or older in the study. The mean age of the group was 62.4 (SD:8.3) years. Most of the participants were female (63.8%). 869 (19.31%) adults were sarcopenia, 446 (9.9%) participants were identified as having mild cognitive impairment, 144 (3.2%) adults were identified as having moderate/severe cognitive impairment. The prevalence of sarcopenia in male was 22.1 and 17.8% in female. The prevalence of sarcopenia in the age group of < 65, 65–74, 75–84 and ≥ 85 were 11.2, 26.5, 50.5 and 65.2%, respectively. The prevalence of sarcopenia in different ethnic groups of Han, Zang, Qiang and Yi were 22.3, 18.2, 11.8 and 34.7%, respectively. An overview of clinical and sociodemographic data, anthropometric measures, cognition, depression, lifestyle factors, and chronic diseases are given in Table 1. Compared to the subjects without sarcopenia, those with sarcopenia were on average older, lower educational degree, living alone, bad marriage status (windowed or divorced), poor sleep quality, lower physical activities (indoor housework or outdoor housework), more smoking, less drinking tea, chronic diseases comorbidities burden, higher prevalence of depression and cognitive impairment (Table 1). And the prevalence of different ethnics varies significantly. While drinking alcohol and the prevalence of anxiety status is not significant between sarcopenia group and non-sarcopenia group (Table 1).

**Table 1:** Comparisons of demographic characteristics, anthropometric measures, life-styles, chronic diseases, depression, and cognitive impairment of the study participants with and without sarcopenia. (*N* = 4500)

Characteristics	No Sarcopenia *n* = 3631	Sarcopenia *n* = 869	T/ χ^2^	*p* value
Age,year,mean(±SD)	61.0 (7.5)	67.9 (8.8)	21.39	<.001
50 ≤ Age<65, No(%)	2475 (88.8)	313 (11.2)	433.88	<.001
65 ≤ Age ≤ 74, No (%)	956 (73.5)	345 (26.5)		
75 ≤ Age ≤ 84, No (%)	192 (49.5)	196 (50.5)		
85 ≤ Age, No (%)	8 (34.8)	15 (65.2)		
Gender No (%)				
Male	1268 (77.9)	359 (22.1)	12.41	<.001
Female	2363 (82.2)	510 (17.8)		
Ethnics			83.15	<.001
Han,No(%)	1506 (77.7)	431 (22.3)		
Zang,No(%)	1009 (81.8)	224 (18.2)		
Qiang,No(%)	926 (88.2)	124 (11.8)		
Yi,No(%)	139 (65.3)	74 (34.7)		
Others,No(%)	56 (76.1)	17 (23.9)		
Education,No(%)			49.17	<.001
No formal education	972 (26.8)	322 (37.1)		
Elementary school	1161 (32.0)	287 (33.0)		
Middle school	787 (21.7)	134 (15.4)		
High school and above	711 (19.6)	126 (14.5)		
Marital status, No (%)			56.84	<.001
Single	23 (0.7)	8 (1.0)		
Married	2965 (86.0)	628 (76.0)		
Divorced	52 (1.5)	15 (1.8)		
Widowed	409 (11.9)	175 (21.2)		
Anthropometric measures				
Grip strength, mean(±SD)	23.0 (8.7)	17.6 (6.8)	19.94	<.001
Gait speed, mean(±SD)	0.88 (0.3)	0.74 (0.3)	13.41	<.001
ASMI, mean(±SD)	6.8 (0.9)	5.7 (0.7)	40.14	<.001
Life-styles				
Living alone No(%)				
Yes	153 (4.2)	57 (6.6)	8.67	.003
No	3478 (95.8)	812 (93.4)		
sleep quality No(%)				
PQSI>5	1595 (46.3)	420 (51.0)	5.90	.015
PQSI≤5	1848 (53.7)	403 (49.0)		
Drinking Tea, No (%)				
Yes	1672 (48.7)	373 (45.5)	2.72	.099
No	1762 (51.3)	447 (54.5)		
Drinking alcohol, No (%)				
Yes	876 (25.5)	195 (23.8)	1.08	.299
No	2559 (74.5)	626 (76.2)		
Smoking history No(%)				
Yes	551 (16.0)	187 (22.8)	21.16	<.001
No	2885 (84.0)	633 (77.2)		
Indoor housework No(%)				
No	952 (27.8)	335 (41.2)	55.80	<.001
Yes	2472 (72.2)	478 (58.8)		
Outdoor housework No(%)				
No	1974 (57.8)	511 (63.1)	7.54	.006
Yes	1441 (42.2)	299 (36.9)		
Number of chronic diseases			4.92	.027
0 or 1, No (%)	934 (25.7)	192 (22.1)		
≥ 2, No(%)	2697 (74.3)	677 (77.9)		
Depressive status, No (%)				
GDS-15<5	2816 (77.6)	639 (73.5)	6.36	.012
GDS-15 ≥ 5	815 (22.4)	230 (26.5)		
Anxiety status, No (%)				
GAD-7<5	2950 (81.2)	708 (81.5)	0.02	.877
GAD-7 ≥ 5	681 (18.8)	161 (18.5)		
Cognitive function, No (%)			92.59	<.001
Complete cognitive function	3023 (88.1)	637 (77.7)		
Mild impairment, No (%)	331 (9.7)	115 (14.0)		
Moderate-severe impairment, No (%)	76 (2.2)	68 (8.3)		

*Note.* Means ± standard deviation was shown. Others = other ethnics including Zhuang, Manchu, Hui, Mongolia, Tujia ethnics. Data are shown using % or mean (standard deviation). *P* values were calculated with chi-squared tests and Student’s t tests for categorical and continuous variables, respectively

Table 2 shows the association between sarcopenia and cognitive impairment using multiple logistic regression in three models. In model 1 which only adjusted age, sex and ethnics, cognitive impairment (mild: OR 1.49, 95% CI 1.16–1.92, *P* = .002; moderate/severe: OR 3.41, 95% CI 2.34–4.97, *P* < .001) was significantly associated with sarcopenia (Table 2). After adding adjusting the marriage status, living alone, life-style factors, physical activities and sleep quality in model 2, results showed that sarcopenia was still significantly associated with mild cognitive impairment (OR: 1.45, 95% CI, 1.12–1.87, *P* = .004) and moderate/severe cognitive impairment (OR: 3.27, 95% CI, 2.23–4.79, *P* < .001) (Table 2). And this is consistent in the fully adjusted model 3 after adding chronic diseases comorbidities and depressive status which showed sarcopenia was significantly associated with mild cognitive impairment (OR: 1.41, 95% CI, 1.10–1.82, *P* = .009) and moderate/severe cognitive impairment (OR: 3.05, 95% CI, 2.08–4.49, *P* < .001). Besides, we analyzed the association between three elements of sarcopenia and cognition function separately. And it shows that cognitive impairment was significantly associated with grip strength, gait speed and ASMI separately with a dosage effect. After gender stratification in fully adjusted model, mild cognitive impairment is not significantly associated with sarcopenia in male, but is still significant in female. While moderate/severe cognitive impairment in male has a higher odds ratio than female (male: OR: 4.84, 95% CI, 2.01–11.62, *P* < .001; female: OR: 2.64, 95% CI, 1.71–4.10, *P* < .001) (Table 3). After age stratification in fully adjusted model, moderate-severe cognitive impairment was significantly associated with sarcopenia in participants less than 85 years old. Mild cognitive impairment was only associated with sarcopenia less than 65 years old (Table 3).

**Table 2:** Multiple regression analysis of sarcopenia and the three factors of sarcopenia (grip strength, gait speed, ASMI) with cognitive impairment among multi-ethnics in the west China communities. (*N* = 4500)

Sarcopenia	Model 1, OR(95%CI), *p* value	Model 2, OR(95%CI), *p* value	Model 3, OR(95%CI), *p* value
Cognitive impairment			
No	1.0(Ref)	1.0(Ref)	1.0(Ref)
Mild	1.49 (1.16–1.92), .002	1.45 (1.12–1.87), .004	1.41 (1.10–1.82), .009
Moderate-severe	3.41 (2.34–4.97),<.001	3.27 (2.23–4.79),<.001	3.05 (2.08–4.49),<.001
Grip strength			
Cognitive impairment			
No	1.0(Ref)	1.0(Ref)	1.0(Ref)
Mild	1.47 (1.19–1.82),<.001	1.41 (1.14–1.75), .002	1.38 (1.11–1.71), .004
Moderate-severe	2.24 (1.51–3.32),<.001	2.11 (1.42–3.14),<.001	2.00 (1.34–2.97), .001
Gait speed			
Cognitive impairment			
No	1.0(Ref)	1.0(Ref)	1.0(Ref)
Mild	1.50 (1.22–1.86),<.001	1.44 (1.16–1.78), .001	1.42 (1.14–1.76), .001
Moderate-severe	1.76 (1.22–2.56), .003	1.66 (1.14–2.42), .008	1.60 (1.09–2.33), .016
ASMI			
Cognitive impairment			
No	1.0(Ref)	1.0(Ref)	1.0(Ref)
Mild	1.33 (1.05–1.68), .017	1.31 (1.04–1.67), .025	1.29 (1.02–1.64), .038
Moderat-severe	2.83 (1.96–4.07),<.001	2.74 (1.89–3.96),<.001	2.60 (1.79–3.77),<.001

*Note. Abbreviations*: *OR* odds ratio, *CI* confidence interval

Model 1: adjusted for age, sex and ethnics

Model 2: adjusted for age, sex, ethnics, marriage status, living alone, life styles (smoking, drinking tea), sleep quality, indoor housework and outdoor housework

Model 3: adjusted for age, sex, ethnics, marriage status, living alone, life styles (smoking, drinking tea), sleep quality, indoor housework, outdoor housework, chronic diseases comorbidities and depressive status

**Table 3:** Association of cognitive impairment and sarcopenia among multi ethnics in the west China communities after gender and age stratification (*N* = 4500)

Variables	Adjusted OR (95% CI)	*P* value
Male		
Cognitive impairment		
Mild	1.46 (0.89; 2.37)	.132
Moderate-severe	4.84 (2.01; 11.62)	<.001
Female		
Cognitive impairment		
Mild	1.39 (1.02; 1.89)	.035
Moderate-severe	2.64 (1.71; 4.10)	<.001
50 ≤ Age<65		
Cognitive impairment		
Mild	1.88 (1.27–2.76)	.001
Moderate-severe	3.66 (2.06–6.53)	<.001
65 ≤ Age ≤ 74		
Cognitive impairment		
Mild	1.11 (0.73–1.69)	0.630
Moderate-severe	2.17 (1.17–4.02)	0.014
75 ≤ Age ≤ 84		
Cognitive impairment		
Mild	1.47 (0.80–2.71)	0.210
Moderate-severe	3.56 (1.31–9.63)	0.013
85 ≤ Age		
Cognitive impairment		
Mild	–	NS
Moderate-severe	–	NS

*Note.* Adjusted odds ratios were computed by adjusting for age, ethnics, marriage status, life-style factors (smoking, drinking tea), living alone, sleep quality, indoor housework, outdoor housework, chronic diseases comorbidities, depressive status; *CI* confidence interval, *OR* odds ratio, *NS* no significant

## Discussion

According to reports from different countries in Asia, the prevalence of sarcopenia varies from 7.8–35.3% depending on different diagnostic instruments, different age and living conditions of the study participants in each study [17]. In our study, the mean age is 62.4 (SD ± 8.3) and the prevalence of sarcopenia is 19.31%, which was similar to a cohort study in Taiwan using the AWGS diagnostic criteria that the prevalence of sarcopenia was 21% in participants aged 75 years and older [18]. Another cohort study in northern Taiwan included 302 individuals (157 men and 145 women) aged 65 years and older, and the prevalence of sarcopenia in that study was 18.6% in elderly women and 23.6% in elderly men using BIA analysis [11]. A previous study in Chengdu found approximately 10% community-dwelling elderly have sarcopenia in a number of 947 residents aged ≥60 years old which is also estimated by the BIA analysis [19]. And the prevalence of sarcopenia was 12.3% in Chinese men and 7.6% in Chinese women aged 70 years and older in Hongkong estimated by dual-energy X-ray absorptiometry [20]. This study found a higher prevalence than Chengdu and Hongkong. The reason might be that most participants were living in the village that were poorer and have lower education level even through the mean age of our study is lower than the previous studies. This shows sarcopenia are happening younger in rural areas. And the difference may also be derived from the geographical and diagnostic criteria for sarcopenia and the difference in cognitive function diagnostic criteria. Besides, our study also found that advanced age with a dose response effect associated with sarcopenia and this is compatible with many other studies [20–22]. Moreover, our study found that the prevalence of sarcopenia in male is higher than in the female. This is not consistent with other previous studies which showed Chinese women may be more vulnerable to severe sarcopenia in old age than men [23]. The reason might be that the mean age of male is older than the female in our study. Besides, our findings revealed the prevalence of sarcopenia in different ethnic groups are different. This might be related with different cultural life-styles and diet habit. Also related with different composition of age and gender. Further research of the ethnic groups and sarcopenia could be done in the future.

Our findings revealed that sarcopenia was significantly associated with cognition impairment in the multi-ethnic western Chinese population, independent of age, gender, ethnics, marriage status, lifestyle factors, indoor or outdoor housework, sleep quality, chronic diseases comorbidities and depression status. This is compatible with several other studies that shows a positive relationship between cognitive impairment and sarcopenia [24–26]. While in the EPIDOS cohort study, sarcopenia was not shown to be associated with cognitive impairment in elderly women [27]. Several possible mechanisms in many studies could explain this association. Firstly, cognitive impairment leads to reduced physical activity and dietary intake, which could trigger excessive muscle loss in older adults, accelerating sarcopenia [28]. Secondly, low-grade inflammation is considered to be associated with both sarcopenia and cognitive impairment. Interleukin-6 (IL-6) and tumor necrosis factor-α (TNF-α) have also been reported as inflammatory parameters, which are the important factors to lead to sarcopenia [29] and the development of cognitive impairment [30]. Third, excessive oxidative stress related to chronic diseases also play important roles in age-related muscle atrophy, interfering with the balance between protein synthesis and breakdown, causing mitochondrial dysfunction, inducing apoptosis, and lead to sarcopenia [31]. While products of oxidative and nitrosative stress accumulate with aging, which is also the main risk factor for cognition impairment [32]. In the end, cognitive impairment reinforces and emphasizes the neuronal changes in the central nervous system leading to changes in the levels and activity of neurotransmitters, which together with the inadequate distribution of oxygen to the brain, lead to a reduction in motor units and in the ability to maintain muscle activation, which might be related to sarcopenia [33]. Thus, there exists a close relationship between sarcopenia and cognitive impairment which share some common mechanisms.

Besides, our study found that the association between sarcopenia with moderate/severe cognitive impairment in male has a higher odds ratio than female which might be related to hormone regulation. And moderate/severe cognitive impairment is positively related to sarcopenia in women and men, while the mild cognitive impairment seems not be related to sarcopenia in male. And a recent study indicated significant associations of sarcopenia and physical frailty with cognitive decline was could be more easier to be found in females [34]. One study explained this sex differences may be partly due to systemic inflammation [35]. A larger sample prospective cohort studies are required to illustrate gender differences. Besides, after age group stratification, it shows that moderate/severe cognitive impairment was significantly associated with sarcopenia nearly in every age group, proving a close relationship between sarcopenia and cognitive impairment. While in the age group over 85 years old, the relationship was not significant anymore might be the reason that there were only 23 participants in that age group which might exist some bias.

Moreover, our study analyzed the association between cognitive impairment and the three elements in the fully regression model and it shows significant association in grip strength, gait speed and ASMI with a dosage effect separately. And this is consistent with recent studies. A study had shown that handgrip strength could be a means of monitoring progression of cognitive decline [36]. And some longitudinal studies found that those with slower walking speeds and a greater decline in speed over time were at greater risk of developing dementia and could predict transitioning from mild to severe cognitive impairment [37, 38]. However, for the element of ASMI, recent study found that Lower-extremity functioning, rather than ASMI, is closely related to multiple cognitive domains [39]. While low muscle mass was found to be predictors of long-term mortality in nonagenarian and centenarian women [40]. Whether ASMI could be used as a predictor of cognitive impairment required more studies.

This is a large study to assess sarcopenia defined by the AWGS criteria and its association with cognitive impairment in multi-ethnic western China, adjusting the analysis for relevant confounders, such as sociodemographic characteristics, marriage status, lifestyle factors, and chronic diseases comorbidities. Nonetheless, this study presents some limitations. It is derived from a cross-sectional study, in such a way that it is not possible to conclude the existence of a causal association between sarcopenia in the participants and the associated factors mentioned here, which is feasible in longitudinal designs. Although regression models were adjusted for many variables, residual confounding is still possible. In addition, chronic disease information, cognition, and depression adjustment come from self-reported questionnaires, and in spite that all the data in the questionnaires have been previously validated, this information should be treated with caution. In the end, we conducted a centralized investigation, not a household survey in which most of the participants who can come by themselves are relatively healthy people. As a result of it, there exists offset in the result of prevalence. However, despite these limitations, this study has enough samples for adjustment of the regression models to major confusion factors of clinical interests. Moreover, it presented similar results to those found in studies with a more robust methodology.

## Conclusions

This study demonstrated that the prevalence of sarcopenia was 19.31% among 4500 multi-ethnic participants aged 50 years old or older in western China and sarcopenia was significantly associated with cognitive impairment with a dosage effect, especially in female. This is a large cross-sectional study in multi-ethnic western China which is significant to further study of sarcopenia in China and filled up the prevalence of sarcopenia in the western Asia which is important in promoting Asia sarcopenia research as Asia is made up of a great number of ethnicities. Moreover, considering the health consequences of sarcopenia is increasingly being recognized, further longitudinal study is required to explore the causal relationship between sarcopenia and cognitive impairment.

## Data Availability

The datasets generated and analyzed during the current study are not publicly available due to this is a newly database which has a lot of important information and we are applying some important projects based on this. But this dataset will be available 2 years later and is also available now from the corresponding author on a reasonable request.

## Abbreviations

ASMI: Skeletal muscle mass index
AWGS: Asian working group for Sarcopenia
BIA: Bioelectrical impedance analysis
EWGSOP: European working group on sarcopenia in older people
GAD-7: Generalized anxiety disorder
GDS-15: 15-item Geriatric depression scale
IL-6: Interleukin-6
OR: Odds ratio
SPMSQ: Short portable mental status questionnaire
TNF-α: Tumor necrosis factor-α
WCHAT: West-China health and aging trend
